# Preclinical Study of a Multi-Layered Antimicrobial Patch Based on Thin Nanocomposite Amorphous Diamond Like Carbon Films with Embedded Silver Nanoparticles

**DOI:** 10.3390/ma13143180

**Published:** 2020-07-16

**Authors:** Tadas Juknius, Indrė Juknienė, Tomas Tamulevičius, Modestas Ružauskas, Ina Pamparienė, Vaidas Oberauskas, Aušrinė Jurkevičiūtė, Andrius Vasiliauskas, Sigitas Tamulevičius

**Affiliations:** 1 Institute of Materials Science, Kaunas University of Technology, K. Baršausko St. 59, LT-51423 Kaunas, Lithuania; ausrine.jurkeviciute@ktu.lt (A.J.); andrius.vasiliauskas@ktu.lt (A.V.); sigitas.tamulevicius@ktu.lt (S.T.); 2 Veterinary Academy, Lithuanian University of Health Sciences, Tilžės St. 18, LT-47181 Kaunas, Lithuania; indre.jukniene@lsmu.lt (I.J.); modestas.ruzauskas@lsmuni.lt (M.R.); ina.pampariene@lsmuni.lt (I.P.); vaidas.oberauskas@lsmuni.lt (V.O.); 3 Department of Physics, Kaunas University of Technology, Studentų St. 50, LT-51368 Kaunas, Lithuania

**Keywords:** DLC, silver, nanocomposite, antimicrobial, *S. aureus*, patch, wound, laboratory animals

## Abstract

A growing number of severe infections are related to antibiotic-resistant bacteria, therefore, in recent years, alternative antimicrobial materials based on silver nanoparticles (NPs) attracted a lot of attention. In the current research, we present a medical patch prototype containing diamond-like carbon nanocomposite thin films doped with silver nanoparticles (DLC:Ag), as a source of silver ions, and an aqueous mass of the gelatin/agar mixture as a silver ion accumulation layer. The DLC:Ag thin films with 3.4 at.% of silver were deposited on synthetic silk employing reactive unbalanced DC magnetron sputtering of the silver target with argon ions performed in the acetylene gas atmosphere. The average size of the silver nanoparticles as defined by scanning electron microscope was 24 nm. After the film deposition, the samples were etched with RF oxygen plasma, aiming at efficient silver ion release in aqueous media from the nanocomposite film. In the patch prototype, a mixture of agar and gelatin was applied in silicone carrier with cavities, acting as a silver ion accumulation layer that further enhanced the antimicrobial efficiency. It was found that the DLC:Ag thin film on the silk after soaking in water for 24 h was able to release up to 4 ppm of Ag. The microbiological experiments using *S. aureus* bacteria were performed with the patch prototype and the silver ion saturated water, demonstrated the inactivation of 99% and 79% of bacteria, respectively. Scanning electron microscopy analysis showed that silver NPs destroyed the bacteria cell and the bacteria affected by Ag ions had spots and perforated cell wall areas with cytoplasm leakage out was obtained. A preliminary preclinical study using the laboratory animals demonstrated that using the patch prototype, the methicillin-resistant *S. aureus* (MRSA)-infected wound on skin surface healed faster compared with control and was able to kill all MRSA bacteria strains in the wound’s bed after 72 h of treatment.

## 1. Introduction

Today, medicine faces a lot of challenges in wound care, related to methicillin-resistant *Staphylococcus aureus* (MRSA) caused infections after simple injuries, like burns or cuts, which were influenced by the extensive use of antibiotics. After the discovery of the first antibiotic penicillin by Alexander Fleming in 1928 [1], it had a strong influence on the increase in survival rate after bacterial infection. With time, the effectiveness of antibiotics decreases because of acquired antimicrobial resistance and multi-resistant strains such as MRSA become a big challenge for today’s medicine. Since the 1960s, infections after skin injuries become difficult to treat due to the increasing number of antibiotic-resistant bacteria like *Staphylococcus* spp., and particularly MRSA. It is estimated that up to 65% of all *S. aureus* infections are caused by MRSA [2]. Bacteria in nature exist as sessile communities on a variety of surfaces [3,4], and the skin of the human body is covered with biofilms of various, usually not pathogenic, bacteria species [5,6]. The immune system took an essential role in the infection pathway. When the immune system weakens, or pathogenic bacteria starts to proliferate, it results in an infection [7]. Certainly there are many causes of delayed wound-healing and not all of them are directly involved in biofilm formation, but it should be noted that, in the case of chronic wounds, the formation of bacteria biofilm for the 60% of wound clinical cases [8], is one of the main factors for the wound transition into a chronic state. The first aid for injured tissues in hospitals, the battle-field, or at home [9,10] for stopping bleeding and contamination with dirt and bacteria [11,12] are bandages and patches. Applied on the wound, they have direct contact with the soft wound tissues, where exudate and nutrients from the blood can nurture bacteria multiplication and formation of the biofilm [7,8]. If the wounds are not dressed timely, the latter processes usually end up as serious infections. A lot of various solutions, gels, creams, and bandages are used in hospitals for the treatment of wounds [11]. The patches are usually made from cotton, but there are some bandages and patches made from hydrogels and other water-absorbing materials. For example, cellulose possessing antimicrobial properties could be a promising material because it is non-toxic, demonstrates good mechanical stability and ability to sustain high content of moisture [13]. Scientific studies confirmed that silver can noticeably improve the antimicrobial properties and speed up healing of the wound [14,15]. Therefore, nowadays the silver-containing patches and bandages are used. Silver can be coated on cotton surface and, when in contact with aqueous media, it releases the silver ions (Ag^+^) [16,17]. Even more advanced patches and bandages for wound care are made from hydrogels [11,12,18].

The antimicrobial mechanisms of the silver-containing materials are still debated. The zero-valent silver (bulk metal) does not demonstrate antibacterial properties [14,19], but after being in contact with aqueous media, the silver metal atoms are released from the silver surface via oxidative dissolution [4], becoming positively charged silver ions. It was reported that Ag^+^ is partially responsible for the antibacterial effect [11,20,21,22]. The typical antibacterial features for silver nano-objects which are due to the controlled release of Ag+ ions were observed in [23]. One of the most popular explanations of the silver ions antimicrobial mechanism is Ag^+^ interaction with the thiol groups in the plasmic membrane [24] and their chemical bonding with the cell surface proteins containing Fe-S groups [25]. According to the suggested photocatalytic mechanism, silver ions can result in the blockage of cell respiration process by activated oxygen molecules and the hydrogen atoms of the thiol groups that are affected by catalytic oxidation reactions [26]; as a result, the two thiol groups, which are covalently linked together by disulfide links (R-S-S-R) and are inactivated [11,15]. Among others, it is suggested that the silver ion can block the electron transport system, which leads to cells death [27]. Silver ions can inactivate bacteria cytoplasmic proteins, block the ATP functions [21], interact the DNA transcription replication processes [20,28,29].

The concentration of the released silver ions is directly related to the sample surface area and time duration of contact with water. Nanomaterials are known to have a high surface to volume ratio; therefore, nanocomposite films with embedded silver nanoparticles (NPs) [30] demonstrate high antimicrobial efficiency [11]. Colloidal NPs can attach to the cell wall and penetrate inside them, interacting with biomolecules and influence their behavior in the cell [11,31]. It is known that NPs can accumulate on the cell wall by attaching to it, and induce the outer membrane destabilization process, resulting in morphological changes such as membrane damage [25] and irregular shape pits on bacteria surface can be detected [31,32]. The importance of the NP’s size dependence was pointed out in [24], where it was obtained that NP’s size determines the antibacterial properties. For example, NPs < 10 nm in diameter can attach to the membrane of the bacteria due to electrostatic force, while larger NPs cannot [21,33]. In short, the mechanism could be described as a mechanical attack to the cell wall by nanoscale aggregates, which causes permanent bacterial cell wall damage as a result—the release of the intracellular content [34].

In summary, the antimicrobial mechanism of Ag NPs is still under discussion, but it is generally accepted that the two already-discussed mechanisms prevail: (i) the mechanical one where NPs directly impose the damage to the cell [31,35] and (ii) the one determined by the Ag ions when they are released from the NPs and interact with the cell structure and molecules inside the cell [36]. Both antimicrobial mechanisms in water media can occur simultaneously and reinforce the antimicrobial effect of silver [11,31,37].

Compared with antibiotic therapies applied to MRSA caused infection treatment, the silver has minor toxicity for the cells, it possesses a wide range of antimicrobial activity mechanisms, and bacteria do not gain the same resistance to silver as the antimicrobial agent, and could prevent MRSA infections or contribute in the healing of difficult clinical cases.

In our work, an original patch prototype containing DLC:Ag thin film as a source of silver ions, and an aqueous mass of the gelatin/agar mixture as a silver ion accumulation layer is studied. The DLC:Ag thin film on synthetic silk was chosen as the biocompatible, durable material, and silver NPs were embedded into the DLC matrix, which provided efficient silver ion release to the aqueous media without any chemical or mechanical remains [38]. The patch was applied for the in vivo and in vitro treatment of *Staphylococcus aureus* bacteria. The antimicrobial efficiency of the patch prototype, are compared with the control measurements employing silver-ion-saturated water and DLC:Ag-soaked bacteria solution, helping to elucidate the pathways of the antimicrobial mechanisms of the proposed patch. The preliminary pre-clinical animal study of the patch prototype was performed using Guinea pigs, where the healing effect of the MRSA-infected wounds was evaluated, and the antimicrobial activity of the patch prototype was investigated.

## 2. Materials and Methods

The bandage prototype was made from the following materials: medical-grade silicone elastomer “A-103” (www.factor2.com, braided line) “Berkley Micro Ice 0.006 mm”, the cellulose fibers (medium, C6288 Sigma, Sigma Aldrich, St. Louis, MO, USA), gelatin (53028 FLUKA, Sigma Aldrich), agar (A1296 SIGMA, Sigma Aldrich), textile (twill weaved synthetic silk, with a weft density of 110 cm^−1^ and a warp density of 100 cm^−1^) coated with DLC:Ag thin film and a crystalline silicon wafer as a substrate for control measurements in every process, respectively.

Materials utilized for the microbiological experiments included: Mueller Hinton agar (Thermo Scientific, Leicestershire, UK), 94 mm-diameter Petri dishes, ethyl alcohol (96%) (AB Stumbras, Kaunas, Lithuania). The bacteria strains were *S. aureus* (ATCC25923) referential strain, and methicillin-resistant *S. aureus* strain—LTSa635—which was isolated from the infected skin of man in Lithuania.

For the animal model, the Guinea pigs were used for patch testing.

The nanocomposite DLC:Ag thin films were deposited employing a DC-unbalanced magnetron with the silver target in acetylene gas atmosphere C_2_H_2_ gas (99.999%). RF plasma etching of the DLC:Ag films was carried out employing Plasma-600-T (JSC Kvartz) device and oxygen gas (99.9%). DLC:Ag chemical composition and silver NP’s size distribution analysis was performed employing SEM Quanta 200 FEG (FEI) with an energy-dispersive X-ray spectrometer (EDS) system Quantax (Bruker). The EDS measurements were performed at 5-keV accelerating voltage, and silver NP analysis was preformed from SEM micrographs employing ImageJ (NIH) software and custom MATLAB (MathWorks) code.

The silver ion release experiments from DLC:Ag coatings was performed in the thermostat (35 °C) for different time durations, and the silver ion concentration in distilled water, after rinsing the synthetic silk coated with DLC:Ag samples from 20 min to 48 h, was carried out using the atomic absorption measurements applying the A Perkin Elmer Model 403 spectrometer. Silver-ion-saturated water was prepared rinsing the pure silver 99.999% pieces (from 2 to 6 mm) for a month at room temperature in purified water. The water was filtered, and the silver ion concentration was measured by applying an atomic absorption spectrometer before preparing the silver-ion-saturated water samples for bacteria experiments.

### 2.1. Preparation of Bandage Prototype

DLC:Ag thin film (3.4 at.% Ag) was deposited on synthetic silk and crystalline silicon wafer substrates. The films were sputtered by a DC-reactive unbalanced magnetron, using pure silver target in acetylene atmosphere. The Ag content in DLC samples was controlled by varying the rates of the feed stock gas flow, magnetron power and voltage. The sputtering duration was 235 s, and argon gas flow into the chamber was 70 sccm, acetylene—21.1 sccm. During the deposition, the magnetron current and voltage were varied from 0.07 to 0.12 A, and 553 to 741 V, respectively. More details on the deposition of nanocomposite thin film can be found elsewhere [39].

DLC:Ag-coated samples were dry-etched for 20 s, (RF plasma; 13.56 MHz) by oxygen plasma in 133 Pa pressure, applying 0.3 W/cm^2^ power, and later on disinfected with UVB in the laminar box for 4 h. DLC:Ag-coated synthetic silk samples (20 × 40 mm) were covered with a thin layer (0.01–0.02 mm) of cellulose fibers with gelatin To remove water and gelatin excess, it was pressed by a stainless steel roller, applying 5 kg/cm^2^ pressure.

The silver ion accumulation layer (SIAL) was prepared applying a mesh-like 3D silicone honeycomb structure. The square shaped hollows were of the size 2 mm × 2 mm separated by 0.3 mm walls, and the mesh thickness was 2 mm. The mesh was manufactured using soft lithography processes, applying the two-component medical grade silicone on the mold and crosslinking for 48 h. After removal of the molded replica, it was washed in ethyl alcohol. The cavities of the mesh were filled with warm suspension (40–45 °C) of gelatin and agar (90%/10% ratio) and stored until they hardened. The structutre of the patch prototype is provided in Section 3.2.2. The patch prototype was prepared in the laminar box.

### 2.2. Microbiological Methods

Current microbiological experiments for the determination of the DLC:Ag thin-film-coated samples and silver-ion-saturated water antimicrobial activity of the referential *S. aureus* (ATCC25923) bacteria were repeated four times in microbiology laboratory applying the spread plate method.

The wound treatment experiments and wound healing observation after contamination of the methicillin-resistant *S. aureus* bacteria strain—LTSa635—were performed in a separated room at the Institute of Microbiology and Virology. The animal model experiments were started on Monday and ended on Friday; the two days (weekend) were without observation. The experiments were repeated four times: on the first working day of the first week, and on the 2nd, 3rd and 4th week. The pathology material (wound fluids) taken from the wound’s bed were transported from the animal storage room to the microbiology laboratory per 10 min and was placed on bacteria nutrition media for the detection of live bacteria number changes.

The experimental results were processed using Excel software. The data were fitted with the exponential and logarithmic functions employing OriginLab (Northampton, MA, USA) software. Detailed functions are provided in Supplementary Table S1.

### 2.3. Spread Plate Method

The spread plate method was used to place (spread applying the swab or a microbiological loop which is bent at an angle of 90°) the bacteria on the agar surface after serial dilutions.

The serial dilution method was used for dense culture reduction, with culture becoming less and less dense to get a more usable concentration of CFU on the Petri dish. By applying this method, each dilution reduces the bacteria concentration 10 times.

After dilution experiments, the 0.1 mL. of bacteria suspension was inoculated onto the agar surface and incubated at 37 °C for 18 h [40]. After the incubation, the surviving bacteria in the single-colony forming unit (CFU) were calculated. The number of live bacteria in suspension can be calculated applying the formula: CFU/mL = (number of colonies × dilution factor)/volume of culture plate [40]. The experiments were repeated four times to ensure statistical reliability.

### 2.4. Tests with the Laboratory Animals

Tests with the Guinea pigs were performed according to the State Food and Veterinary Service permission (Nr. G2-30) in Lithuanian University of Health Sciences, Veterinary Academy. Guinea pigs were selected for the experiments, which helped to obtain all healing processes during the short period-of-time, [41]. Laboratory animals were placed in special cages with small shelters to hide in, and 24 h access to dry special food and water.

During the test procedures, the animal’s fur on the dorsal area was shaved, and the skin was disinfected with 70% of ethyl alcohol. Then, the top skin surface (epidermis) was incised from 26 to 32 mm using the tip of the scalpel, and patch prototype samples were stuck to the skin. Moreover, the special waistcoat made for animals was used to prevent the removal of the testing material. The patch healing effect was evaluated every day and it was sealed for 4 days, until the wound healed (contracted). The infected wound experiments were performed applying the *S. aureus.* bacteria (pathogenic MRSA). During the experiment, after a minor skin cut (up to 34 mm in length), the small drop (0.5 Mf of 20 μL) of bacteria suspension was instilled to the wound and the patch was sealed. The microbiological samples from the tissues (using swab), were taken every day. The experiments were performed with two laboratory animals per experiment, i.e., four patch variations were tested with two animals. The redness, swelling, capillary net growth, wound diameter changes and bacterial CFU inside the wound were registered for the wound-healing evaluation. The healed wound result showed that at least 90% of the wound’s surface was re-epithelized [42].

## 3. Results

### 3.1. Deposition of DLC:Ag Thin Film on Silk

**Figure 1:**
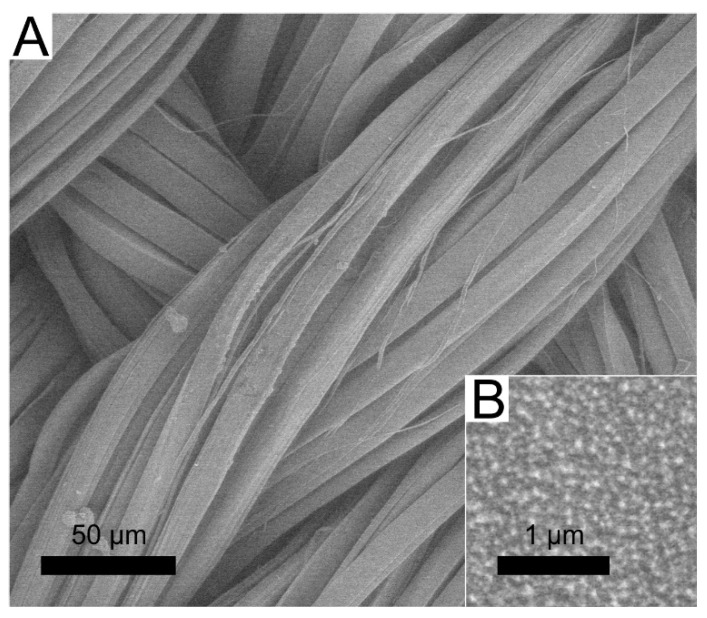
SEM micrograph of the synthetic silk cloth coated with DLC:Ag thin film (**A**), close up view of the film (**B**).

The deposition conditions and the resulting silver content in the DLC:Ag nanocomposite thin film, for the current study were selected based on the best antimicrobial activity of the nanocomposite determined in our previous work [30]. The 3.4 at.% Ag concentration in the diamond-like carbon film sputtered on a synthetic silk cloth was obtained from the measurement of the witness silicon substrate sample. Bearing in mind that, for particular nanocomposite films, plasma-processing is important [30] for the antimicrobial activity, an RF oxygen plasma etching (50 W/cm^2^, 20 s duration) was carried out. The surface morphology of the used synthetic silk cloth and nanoparticles sputtered on the surface are depicted in Figure 1. From the scanning electron microscope (SEM) image analysis, it was determined that the average size of silver nanoparticles was 23.7 nm (±9.6 nm) and the size of silver nanoparticles correspondingly varied from 3 to 63 nm.

### 3.2. Antimicrobial Properties

Aiming to estimate the ion release efficiencies and elucidate their resulting antimicrobial properties, the range of experiments was carried out: (i) DLC:Ag-coated cloth was soaked in the aqueous bacterial solution, (ii) the whole patch (including an aqueous mass of the gelatin/agar mixture, as a silver ion accumulation layer) was soaked in the bacterial solution, and finally (iii) laboratory animal testing with the patch prototype was performed. In all cases, the antimicrobial tests with the referential *S. aureus* bacteria were performed and the experimental results were compared with the control sample. In addition to the DLC:Ag studies, we have performed experiments on the silver-ion-saturated water interaction with the bacterial suspension (iv).

#### 3.2.1. DLC:Ag Film

**Figure 2:**
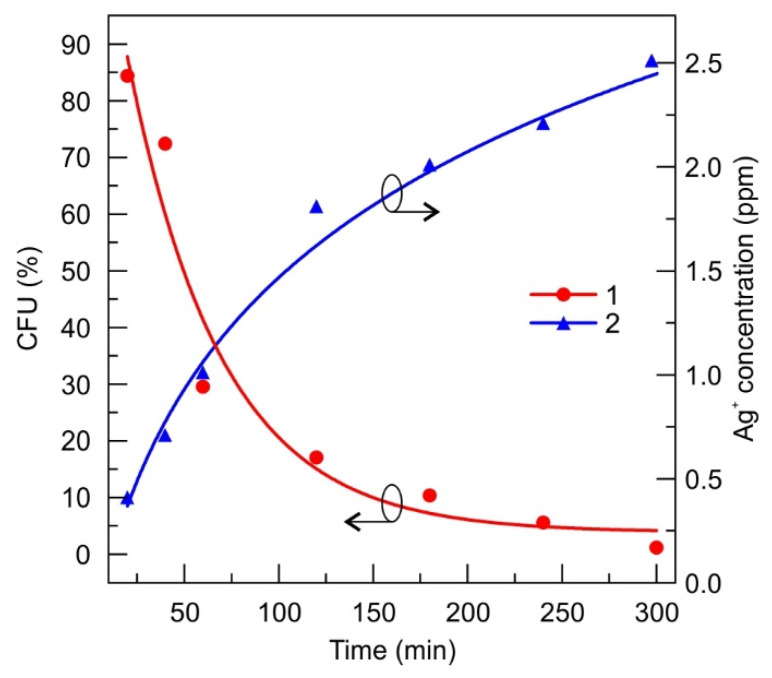
Number of colony-forming units (CFUs), normalized to the control sample, indicating antimicrobial properties of the RF oxygen plasma-processed DLC:Ag film on silk soaked in the bacterial solution (1) and the corresponding evolution of silver ion concentration in time (2). The 0.5 Mf of *S. aureus* bacteria suspension was diluted 10^−5^ and the and active contact surface of the cloth was 6 cm^2^/mL. The curves were fitted with the exponential (1) and logarithmic (2) functions. The fit parameters are summarized in Supplementary Table S2.

The diamond-like carbon with the embedded silver-NP-coated silk sample of 2 × 3 cm in size was tested for antimicrobial activity soaking in the referential *S. aureus* bacteria suspension of 0.5 Mf concentration for different time durations, from 20 to 300 min. The bacteria suspension after soaking of the silk cloth without the nanocomposite film was utilized as a control one. The numbers of colony forming units (CFUs) grown after different times of bacteria solution being in contact with DLC:Ag coated silk cloth are tabulated in the Supplementary Table S1, while the normalized CFU values are depicted in Figure 2. The higher CFU number, likewise a higher percent of CFU, indicates lower antimicrobial efficiency. The slight growth of CFU in the case of the control sample (see Table S1) could be attributed to the natural bacterial multiplication throughout the experiment. The antimicrobial experiments are related to the silver ion release rate, that was estimated over the same periods and determined from the atomic absorption measurements of bacteria-free water where the soaking of DLC:Ag-coated cloth (see Figure 2, “2“) took place.

#### 3.2.2. Antimicrobial Patch Prototype

**Figure 3:**
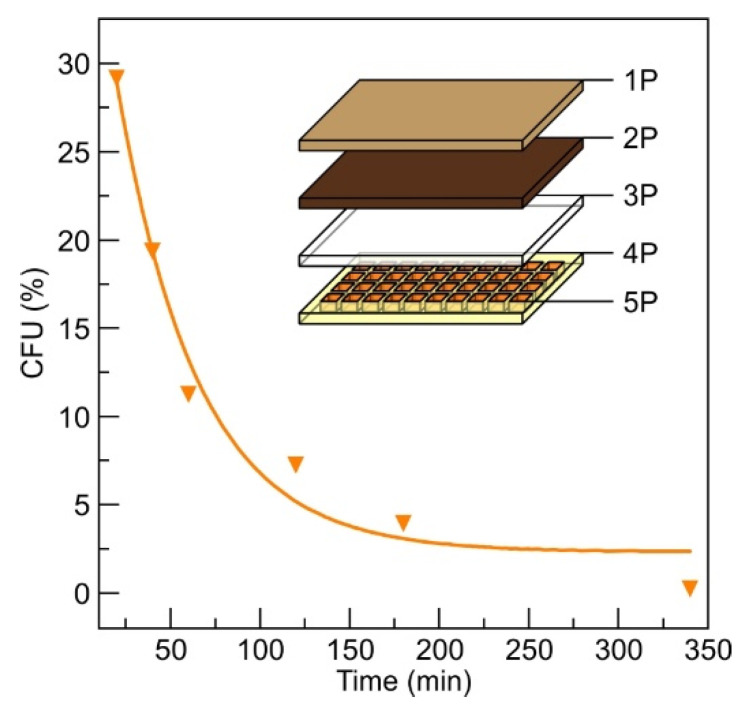
The antimicrobial results of a patch prototype using referential *S. aureus* bacteria of 0.5 Mf. The prepared prototype was soaked in thermostated bacteria suspension at 35 °C temperature. The fit parameters of the exponential curve are provided in Supplementary Table S2. The inset depicts the structure of the patch prototype that consists of the sticky medical film (1P), the synthetic silk coated with DLC:Ag film (2P), cellulose layer (3P), silicon honeycomb carrier structure (4P), gelatin/agar mixture in honeycomb cavities (SIAL) (5P).

The antimicrobial performance of a patch prototype containing 6 cm^2^ silver ion accumulation layer (SIAL, see inset of Figure 3) made from the mixture of gelatin and agar was soaked in the same concentration bacteria solution, as discussed in Section 3.2.1. The obtained antimicrobial performance is depicted in Figure 3. A stronger and faster antimicrobial effect compared with the DLC:Ag thin-film-coated synthetic silk cloth was observed. The SIAL layer of the proposed patch prototype dissolved and spread out the accumulated silver ions into the bacteria suspension at 35 °C resulting in a faster reduction in bacteria population by 70.7% after the first 20 min compared to 15.6% in the case of a single-patch component, i.e., DLC:Ag on silk. As the presence of gelatin and agar in the solution restricted the use of the atomic absorption measurements for determination of the silver release dynamics, additional experiments were carried out with the silver-enriched water containing known concentrations of silver ions.

#### 3.2.3. Silver-Ion-Saturated Water

**Figure 4:**
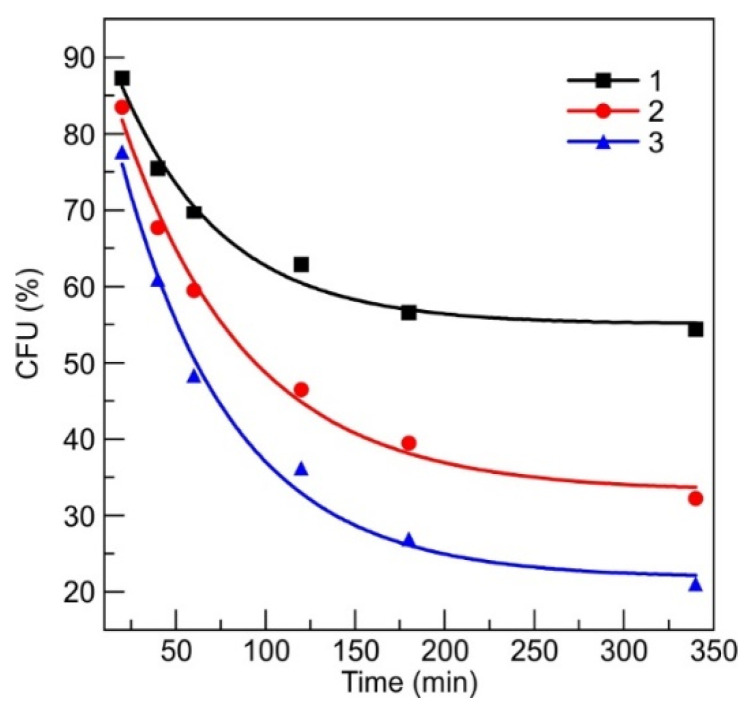
The evolution of antimicrobial activity of silver ion (Ag^+^) rich water of different Ag concentration: “1”—(1 ppm), “2”—(3 ppm), “3”—(4 ppm) in concentration. The fit parameters of the exponential functions are provided in Supplementary Table S2.

Silver-ion-saturated water was prepared by soaking the metallic silver in pure water for 1 month. The concentration of silver was estimated again from the atomic absorption measurements. The stock solution was diluted to 2, 6 and 8 ppm of silver and was mixed with referential *S. aureus* bacteria suspension 1:1 (diluted to 0.5 Mf), and stored for different durations of time ranging from 20 min to 340 min. After mixing, the silver ion concentration was diluted and became 1-3-4 ppm, respectively. The CFU after 20 min decreased from 12.7% to 14.5%, for 1 to 4 ppm, respectively (Figure 4). The actual numbers of the CFUs registered are summarized in Supplementary Table S3.

### 3.3. Microscopic Analysis of Silver-Ion-Treated Bacteria

**Figure 5:**
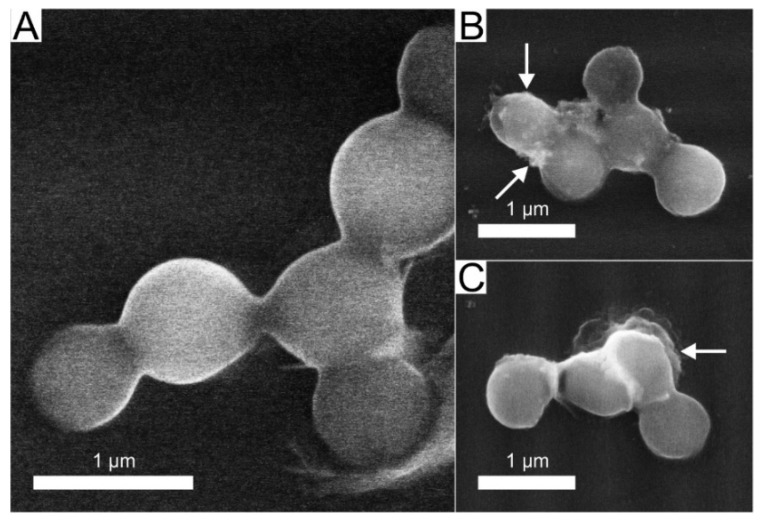
SEM micrographs of silver-treated and untreated *S. aureus* referential bacteria: (**A**)—control (intact), (**B**,**C**)—treated bacteria with silver ions (300 min, 4 ppm), damaged cell walls and cytoplasm leak out observed, are marked with arrows.

After SEM analysis of intact and silver treated bacteria (Figure 5) it was determined that silver ions have a noticeable effect on bacteria cell walls. The SEM micrographs revealed that after exposure of the bacterial solution for 300 min in a colloidal solution of silver with a silver ion concentration of 4 ppm, bacteria cell wall damage took place, identified as visible spots on the surface, as well as leaks out of the cytoplasm (indicated by arrows Figure 5B,C. One can state that the silver ions are efficient in destroying the cells, as a big number of ruptured bacteria cells were found during the SEM analysis.

### 3.4. Short Preclinical Study with the Animal Model

During the preclinical study, the control patch (identical structure to the prototype—except for the DLC:Ag layer) and the proposed prototype patch with DLC:Ag and SIAL (Figure 3) were tested with the Guinea pigs. A preliminary study of patch stability was also performed. The tested patch was packed in a vacuum-sealed polyethylene bag for 6 months and stored at room temperature, and no visible changes were observed in color or consistency. The antimicrobial activity against all tested bacteria strains did not change after the storage time; no allergic skin reaction or inflammation signs were obtained after application to the healthy skin of Guinea pigs.

#### 3.4.1. Wound Treatment with a Control Patch

During the 3 days of treatment, the wound length reduced by 6 mm, inflammatory signs like redness and tissue swelling were observed on the first day, but on the second and third days, these signs reduced. Blood vessel net formation was obtained only on day two (see Table 1, patch 1). The wound was not contracted; it remained open on day 3.

**Figure 6:**
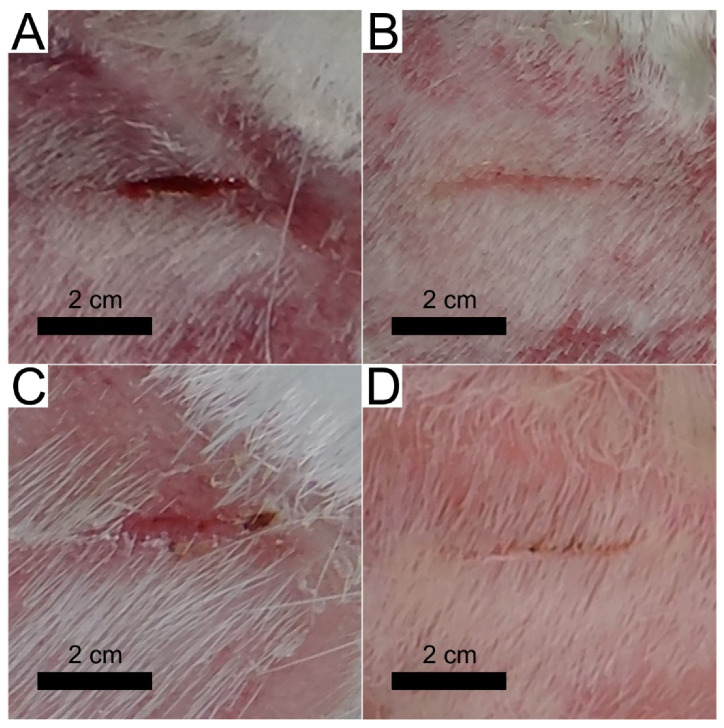
Camera images of the Guinea pig MRSA-infected wound treatment with the control patch (**A**,**B**) and proposed path (**C**,**D**) after 2 days (**A**,**C**) and 4 days (**B**,**D**) of treatment.

The control patch tests with MRSA infected wounds showed the inflammation signs in wound tissues after 24 h, which were registered as the tissues redness and swelling. The 180 CFU of *S. aureus* bacteria were found after microbiological testing. After 48 h, the CFU number was 128 CFU (reduced 28.9%) (see Table 1, patch 2), the swelling was reduced, but the wound bed was a deep red color, and small vessel’s regeneration was obtained. At the third day inflammatory signs reduced, wound length contracted by 4 mm, and 76 CFU (42.2% of CFU survived) were registered. The images after 2 and 4 days of MRSA-infected wound treatment using control patch are depicted in Figure 6.

#### 3.4.2. Wound Treatment with a Patch Prototype

Using the patch prototype containing all layers (as depicted in the inset of Figure 3), after 24 h no strong inflammation signs such as swelling or strong redness were obtained. The healing tissues were light pink, with small growing vessels in the wound’s bed; no scab formation or damaged tissues after patch removal were obtained. After 48 h, the wound tissues were a dark pink color, and the blood vessel’s net regeneration was obtained. On the third day, the wound length contracted by 7 mm, the wound’s length/depth ratio was reduced, and the regenerated light soft pink tissues were obtained.

The patch prototype testing with the MRSA-infected wound showed a reduction in CFU at 1 day by 49.5% comparing with the control tests, and the minimal swelling and redness signs were obtained comparing with the control. After 48 h, the CFU number inside the wound, reduced by 70.6% and wound contracted 2 mm. On day three, no CFU was found, the wound’s length/depth ratio was reduced, and the wound contracted by 4 mm. The strongest healing effect, as expected, was observed for bacteria-free wounds; the length of the wound reduced more compared with MRSA infected one. On day 4, the infected and uninfected wounds contracted, the fur partially covered the patch-treated skin area, and after 5 days of treatment, only small signs of wounds were found in both cases. The images after 2 and 4 days of MRSA-infected wound treatment using patch prototype and control tests are depicted in Figure 6.

## 4. Discussion

According to our results, DLC:Ag nanocomposite demonstrates efficient antimicrobial properties that have been already proved by many researchers [4,39,43], including our previous studies [30]. Moreover, we have shown that this nanocomposite layer can be efficiently explored in a patch as an efficient source of Ag ions. The patch prototype construction [44], based on the use of DLC:Ag nanocomposite film [30], has shown the expected antimicrobial performance that was confirmed in-vitro and in-vivo studies. It should be mentioned that in vitro studies have demonstrated that the use of the SIAL layer ensures efficient instantaneous silver ion release when applied at body temperature. The antimicrobial results of the patch prototype (see Figure 3) demonstrated a much stronger antibacterial effect compared to the DLC:Ag film on silk alone. For example, the number of CFUs decreased to below 30% for the patch, while in the case of the nanocomposite film alone, up to 85% of CFUs were registered. In principle, the silver ion release from the DLC:Ag nanocomposite thin film could be considered as a diffusion from the infinite source, because the concentration of silver ions in water versus time continuously increases (Figure 2) and the antimicrobial effect is not instant, even though, after a certain time, very few CFUs are left. In the case of the patch prototype containing SIAL, the process is more likely due to the diffusion from the finite source with a high concentration of the diffusive species. Answering the natural question of whether (i) the critical silver ion concentration is necessary or (ii) the time of the bacteria exposure to the Ag^+^ is the predominant factor of the antimicrobial effect, we have performed additional experiments with the known fixed silver concentration solutions. The number of silver ions $N_{Ag}$ per milliliter was calculated according to [28]

(1) $$N_{Ag}=\frac{C_{Ag}\;\mathrm{ppm}\times 10^{-6}\left(g\right)}{1\;\mathrm{mL}}\times \frac{N_{A}}{mole\;Ag}$$

where $C_{Ag}$ is the silver ion concentration in parts per million (ppm), $N_{A}$ is Avogadro number (6.02 × 10^23^ mol^−1^), the molar mass of silver is 107.86 (g/mol).

According to the Equation (1), the 4 ppm in 1 mL Ag saturated water contained 2.23 × 10^16^ silver ions. It is known that 0.5 Mf of bacteria suspension contains 1.5 × 10^8^ CFU [45,46], then one bacteria is treated by $2.23\times 10^{16}/1.5\times 10^{8}=1.49\times 10^{8}$ of silver ions, and when Ag ions contain the 1 ppm in solution, 2 ppm, and 3 ppm, it resulted in 3.72 $\times 10^{7}$, 7.44 $\times 10^{8}$ and 1.11 $\times 10^{8}$ ions per bacteria, respectively. According to [28], most pathogenic organisms are killed at silver ion concentrations of 5–40 parts per million (ppm), with some resistant organisms, including methicillin-resistant *Staphylococcus epidermis*, killed at 60 ppm. In our case, registering the kinetics of killing of pathogenic organisms, we can see (Figure 2) that the antimicrobial properties of silver ions can be observed even in a lower concentration region (2.5 ppm). According to Figure 4, the rate of decrease in CFU versus time, as it can be expected, depends on the concentration of silver nanoparticles and permanent release of silver ions from the patch allows the observation of kinetics (or dependence of the rate of CFU) of the process.

**Figure 7:**
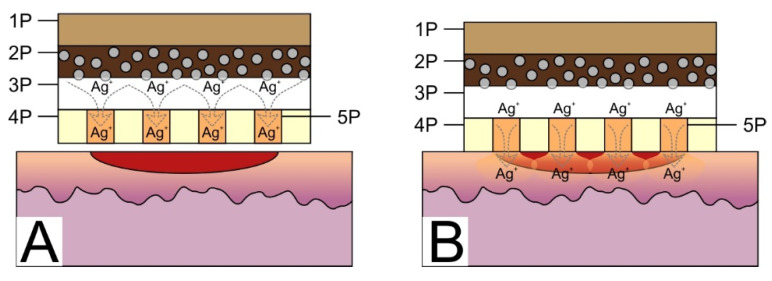
The working principle of the patch prototype. (**A**) Initial Ag^+^ accumulation in the agar/gelatin after application on DLC:Ag-coated silk sample (2P), the silver ions start to diffuse from silver NPs (indicated as grey circles in 2P) through the cellulose membrane (3P) into an agar/gelatin layer (5P) (SIAL). (**B**) When the bandage is applied on wounded skin the gelatin/agar (gel) in the SIAL layer warms up, the gel in silicone honeycomb structure (4P) melts and silver ions can diffuse towards the wound’s tissues (indicated as red half ellipse).

The antimicrobial testing revealed that the initial decrease of CFU resulted in 90–75% of CFUs that are comparable to the results of the DLC:Ag film, only, in the latter case, a smaller Ag^+^ concentration of 0.4 ppm after 20 min was registered. Investigation over longer periods reveals way more expressed differences in the antimicrobial performance on the initial silver concentration in the water where the numbers in CFUs range from 60% to 20%, for 1 and 4 ppm, respectively. This implies that, for the investigated concentration ranges, the silver content does not demonstrate the major role over the short periods, while, in the long run, the influence of the concentration shows up but the antimicrobial effect is not that strong, like in the case of the DLC:Ag. The main reason for this is probably that the DLC:Ag can release the ions constantly, and in the case of the water solution, the number of ions is fixed. The proposed patch containing SIAL brings the advantages of both investigated cases: it provides the momentary release of the silver ions that results in the antimicrobial efficiency of a similar strength like DLC:Ag over longer the timescales necessary to reach the concentration. Moreover, the DLC:Ag that is in contact with SIAL continues to release the Ag^+^ even when SIAL is depleted or dissolved. The proposed mechanism is summarized in Figure 7.

Moreover, the prepared bandage prototype had anti-stick properties which were determined during the experiments with animals, and these anti-stick properties were ensured by SIAL embedding into holey silicone carrier membrane. The honeycomb cavities filled with a gelatin-agar mix saturated with silver ions resulted in a high Ag ion release speed per first 24 h, and continued Ag ion release over several days.

The low contact surface (between the net partitions and wound tissues) reduced the ability to stick to the wound surface, and melted gelatin/agar gel increased these properties even more. Other similar studies with wet bandages, made from hydrogels containing the anti-stick properties, showed similar results but dressings with more adherent contact to wound surface had less good animal-testing results [14,42], as they were obtained in the tests with the proposed patch.

The preclinical study of the patch prototype using an animal model has confirmed the faster contraction of the wound. Moreover, it demonstrated antimicrobial performance against MRSA bacteria when, on day 3, no CFU was found.

## 5. Conclusions

1. The DLC:Ag thin film coated on synthetic silk fabric, oxygen plasma etched and containing 3.4 at.% Ag, with a size of silver nanoparticles (NPs) from 3 to 63 nm and an average size of 23.7 nm, was able to release 4 ppm of silver ions after 24 h of soaking in water, and inactivated 83% of *S. aureus* (0.5 Mf) bacteria per 120 min, while silver-ion-saturated water of 4 ppm, inactivated only 64.1% of bacteria;
2. After *S. aureus* bacteria treatment with silver ions (4 ppm concentration in bacteria suspension), 76% of bacteria were inactivated. Scanning electron microscopy analysis showed that silver NPs destroyed the bacteria cell and the bacteria affected by Ag ions had spots and perforated cell wall areas, where cytoplasm leakage out was obtained;
3. The patch prototype was successfully prepared by applying the DLC:Ag thin-film-coated synthetic silk fabric, which contained the 3.4 at.% Ag. The special cellulose membrane and silver ion accumulation layer were sewed layer by layer on thin-film-coated synthetic silk. The patch demonstrated stable properties for 6 months when stored at room temperature. The prototype increased the healing speed of infected wounds, killed the MRSA inside the wound with more than 50% efficiency per day (completely killed after 3 days), sustained the anti-stick to wound properties and reduced the scar marks.

## 6. Patents

Tadas Juknius. Smart Patch, PCT WO 2018/002817.

## Figures and Tables

**Table 1:** The wound-healing observation: 1—control patch on the uninfected wound; 2—control on methicillin-resistant S. aureus (MRSA)-infected wound, 3—patch prototype on uninfected wound. 4—patch prototype applied on MRSA-infected wound. Numbers represent the values together with their standard deviations, “y”—yes, “n”—no.

Patch No.	Wound Bed Length (mm)			Capillary Net Growth			Swelling			Redness			Bacteria CFU		
Day	1	2	3	1	2	3	1	2	3	1	2	3	1	2	3
Patch 1	31 ± 3	30 ± 2	25 ± 1	n	y	n	y	n	n	y	y	n	-	-	-
Patch 2	30 ± 3	28 ± 1	26 ± 1	n	y	n	y	n	n	y	y	n	184 ± 10	128 ± 8	76 ± 11
Patch 3	26 ± 2	22 ± 1	19 ± 2	y	y	n	n	n	n	n	n	n	-	-	-
Patch 4	32 ± 2	30 ± 2	28 ± 1	y	y	n	n	n	n	n	n	n	91 ± 5	54 ± 6	0

## Supplementary Materials

The following are available online at https://www.mdpi.com/1996-1944/13/14/3180/s1, Table S1: Antimicrobial properties of RF oxygen plasma-processed DLC:Ag film on silk containing 3.4 at.% of Ag. The dilution of bacteria (0.5 Mf) was 10^−5^ and active contact surface was 6 cm^2^/mL. Control test was performed with synthetic silk fabric 6 cm^2^/mL. The samples were thermostated at 35 °C temperature, Table S2: Fit parameters of the CFU (Figure 2) using equation *y* = *y*_0_ + *A*exp(*t/τ*) where *y* is CFU, *t* is the time, *y*_0_ is the offset, *A* is the initial value, R_0_ = 1/*τ* is the decay rate and *τ* is the decay constant) and silver ion concentration using equation *y* = ln(*a* + *bt*) (*y* is silver ion concentration, *t* is the time, *a* and *b* are fittable parameters). *χ*^2^ and *R*^2^ are goodness of the fit. Std.E. stands for Standard Error, Table S3: Antimicrobial properties of silver saturated water containing three different silver ion concentrations: 1 ppm, 3 ppm and 4 ppm. The dilution of bacteria was 10^−5^. Control test was performed with bacteria solution without silver. The samples were thermostated at 35 °C temperature.
