# Supplementary material for: Preclinical Study of a Multi-Layered Antimicrobial Patch Based on Thin Nanocomposite Amorphous Diamond Like Carbon Films with Embedded Silver Nanoparticles
Source: Materials (Basel). 2020 Jul 16;13(14):3180. doi: 10.3390/ma13143180 (PMC7412193; doi:10.3390/ma13143180)
Supplement: Supplementary file 1 [file materials-13-03180-s001.pdf]

materials

Article

Preclinical Study of a Multi-Layered Antimicrobial Patch Based on Thin Nanocomposite Amorphous Diamond Like Carbon Films with Embedded Silver Nanoparticles

Tadas Juknius [1,2,*], Indrė Juknienė [2], Tomas Tamulevičius [1,3,*], Modestas Ružauskas [2], Ina Pamparienė [2], Vaidas Oberauskas [2], Aušrinė Jurkevičiūtė [1], Andrius Vasiliauskas [1] and Sigitas Tamulevičius [1,3]

1 Institute of Materials Science, Kaunas University of Technology, K. Baršausko St. 59, LT-51423 Kaunas, Lithuania; ausrine.jurkeviciute@ktu.lt (A.J.); andrius.vasiliauskas@ktu.lt (A.V.); sigitas.tamulevicius@ktu.lt (S.T.)
2 Veterinary Academy, Lithuanian University of Health Sciences, Tilžės St. 18, LT-47181 Kaunas, Lithuania; indre.jukniene@lsmu.lt (I.J.); modestas.ruzauskas@lsmuni.lt (M.R.); ina.pampariene@lsmuni.lt (I.P.); Vaidas.Oberauskas@lsmuni.lt (V.O.)
3 Department of Physics, Kaunas University of Technology, Studentų St. 50, LT-51368 Kaunas, Lithuania
* Correspondence: tadasjuknius@inbox.lt (T.J.); tomas.tamulevicius@ktu.lt (T.T.); Tel.: +370-696-83-975 (T.J.); +370-662-26-308 (T.T.)

Table 1. Antimicrobial properties of RF oxygen plasma-processed DLC:Ag film on silk containing 3.4 at.% of Ag. The dilution of bacteria (0.5 Mf) was 10^{-5} and active contact surface was 6 cm^2/ml. Control test was performed with synthetic silk fabric 6 cm^2/ml. The samples were thermostated at 35 °C temperature.

Time (min)	Control Sample (Synthetic Silk Fabric)			DLC:Ag of Synthetic Silk		
	CFU (count)	CFU (%)	Ag^+ conc. (ppm)	CFU (count)	CFU (%)	Ag^+ conc. (ppm)
20	488	100		412	84.4	0.4
40	492	100		356	72.4	0.7
60	493	100		146	29.6	1.0
120	504	100	0	86	17.1	1.8
180	502	100		52	10.4	2
240	504	100		28	5.6	2.2
300	505	100		6	1.2	2.5

Table 2. Fit parameters of the CFU (Figure 2) using equation $y = y_0 + A\exp(t/\tau)$ where y is CFU, t is the time, y_0 is the offset, A is the initial value, $R_0 = 1/\tau$ is the decay rate and τ is the decay constant) and silver ion concentration using equation $y = \ln(a + bt)$ (y is silver ion concentration, t is the time, a and b are fittable parameters). χ^2 and R^2 are goodness of the fit. Std.E. stands for Standard Error.

Curve No.	y_0	y_0 Std.E.	A	A Std.E.	R_0 (min^{-1})	R_0 Std.E.	χ^2	R^2
Figure 2 '1'	3.91	6.06	125.47	21.83	−0.0201	0.0063	82.041	0.927
Figure 3	2.33	1.59	41.54	5.63	−0.0223	0.0052	4.414	0.962
Figure 4 '1'	55.09	1.64	44.23	3.98	−0.0176	0.0033	3.591	0.977
Figure 4 '2'	33.18	2.24	64.73	3.93	−0.0143	0.0022	5.040	0.986
Figure 4 '3'	21.82	2.39	74.53	4.94	−0.0159	0.0024	6.644	0.986
**Curve No.**	a	a Std.E.	b	b Std.E.			χ^2	R^2
Figure 2 '2'	0.72	0.14	0.036	0.002			0.010	0.988

Table 3. Antimicrobial properties of silver saturated water containing three different silver ion concentrations: 1 ppm, 3 ppm and 4 ppm. The dilution of bacteria was 10^{-5}. Control test was performed with bacteria solution without silver. The samples were thermostated at 35 °C temperature.

Time (min)	Control		1 ppm		3 ppm		4 ppm	
	CFU (count)	CFU (%)	CFU (count)	CFU (%)	CFU (count)	CFU (%)	CFU (count)	CFU (%)
20	498	100	435	87.3	416	83.5	385	77.3
40	502	100	379	75.5	340	67.7	304	60.6
60	508	100	354	69.7	302	59.4	244	48
120	512	100	322	62.9	238	46.5	184	35.9
180	534	100	302	56.6	211	39.5	142	26.6
340	540	100	294	54.4	174	32.2	112	20.7

© 2020 by the authors. Submitted for possible open access publication under the terms and conditions of the Creative Commons Attribution (CC BY) license (http://creativecommons.org/licenses/by/4.0/).
